# HPV Vaccine Communication and Administration for the Prevention of Oropharyngeal Cancer in Dental Primary Care: Perspectives of Professionals and Students—A Systematic Review

**DOI:** 10.3390/vaccines13030242

**Published:** 2025-02-26

**Authors:** Kenneth Sik-Kwan Chan, Tin-Shun Titan Mak, Ollie Yiru Yu, Victor Ho-Fun Lee, Chun-Hung Chu, Siu-Chee Sophia Chan, Horace Cheuk-Wai Choi

**Affiliations:** 1 Faculty of Dentistry, The University of Hong Kong, Hong Kong, China; 2 Department of Clinical Oncology, School of Clinical Medicine, Li Ka Shing Faculty of Medicine, The University of Hong Kong, Hong Kong, China; 3 School of Nursing, Li Ka Shing Faculty of Medicine, The University of Hong Kong, Hong Kong, China; 4 School of Public Health, Li Ka Shing Faculty of Medicine, The University of Hong Kong, Hong Kong, China; 5 Laboratory of Data Discovery for Health (D24H), Hong Kong Science Park, Hong Kong, China

**Keywords:** dental professionals, dental students, human papillomavirus, HPV, vaccines, oropharyngeal cancer, primary healthcare

## Abstract

**Background/Objectives**: The rising prevalence of HPV-associated oropharyngeal cancer (OPC) presents a significant concern, prompting dental professionals to play an increasingly vital role in HPV vaccination and prevention within primary healthcare. This study aimed to assess the current knowledge, attitudes, and practices of dental professionals and students regarding HPV, the HPV-OPC association, and HPV vaccine communication and administration in dental settings to pinpoint areas for improvement and develop targeted interventions. **Methods**: This study involved a literature search in PubMed/MEDLINE, Embase, and Scopus for research outputs published from 1 January 2006 to 31 December 2024. Eligible studies examined the knowledge, perceptions, and behaviors of dental professionals and students regarding HPV and HPV-OPC. The Risk of Bias Tool was used to evaluate the bias risk in all included studies **Results**: Forty-two studies with a low bias risk were analyzed. While general HPV knowledge was evident in both dental practitioners and students, deficiencies in understanding HPV-OPC and vaccination were identified. Only 9% of dental practitioners discussed HPV vaccination, but future students showed greater willingness (40–80%) to engage in these discussions. Among dental professionals, common barriers included discomfort and a lack of confidence in discussing HPV vaccination. Attitudes towards administering the HPV vaccine varied between dental practitioners and students, with an interest in training programs for readiness. Liability concerns were highlighted as a significant barrier for both groups, impacting their confidence in vaccine administration. **Conclusions**: The findings highlight the need for strategies and areas to enhance knowledge and confidence in discussing HPV vaccines in dental primary healthcare settings, offering valuable insights for researchers and policymakers to plan programs that enhance the readiness of dental professionals to administer HPV vaccines.

## 1. Introduction

Human papillomavirus (HPV) is a prevalent sexually transmitted infection and poses a significant public health concern. HPV infection is implicated in a substantial number of cancer cases worldwide, affecting both women and men and leading to diseases such as cervical cancer, oropharyngeal cancer (OPC), and anal cancer [1]. OPC, once predominantly linked to alcohol and tobacco use, is now increasingly associated with HPV infection, particularly in high-income countries, where its incidence is rapidly escalating [2]. HPV infection has become a major risk factor for OPC, representing one-third of the total cases globally and even higher proportions in countries like the US and the UK [3]. The incidence of HPV-associated OPC (HPV-OPC) is projected to continue its upward trend in the years ahead [2]. Notably, HPV-OPC is more prevalent in males, with a fourfold higher occurrence compared to females [2]. This shift in the etiology of OPC highlights the importance of addressing HPV infection and vaccination as crucial strategies in preventing this increasingly prevalent form of cancer.

HPV vaccination stands as the primary prevention method in the realm of HPV and HPV-associated cancers, proving effective in safeguarding against oncogenic types 16 and 18, which account for 80% of HPV-associated cancers and OPC [2]. HPV vaccines provide the highest protection to vaccinees prior to HPV infection. Individuals are advised to be vaccinated in adolescence, before becoming sexually active. In 2021, the US Food and Drug Administration expanded its approval of the nonavalent HPV vaccine to include the prevention of HPV-OPC [4]. Given the increasing prevalence of OPC, enhancing the HPV vaccination rates and involving primary healthcare providers in discussions about the HPV vaccine with their patients and its administration is crucial.

The evolving role of dental professionals in primary healthcare encompasses not only providing dietary advice and aiding in smoking cessation with counseling and referrals, but also potentially recommending and administering the HPV vaccine to boost vaccination rates [5,6,7,8]. Dental professionals, including dentists and dental hygienists, as primary healthcare providers, have the opportunity to reach a wide patient base, with a significant percentage of adolescents regularly visiting dental offices. Leveraging their position, dental professionals can offer counseling and personalized feedback connecting oral health to the impact of health behaviors during consultations [6,9]. Amid the COVID-19 pandemic, several states in the US enlisted dentists and qualified dental students as vaccinators to enhance the vaccine workforce, facilitating vaccine education and administration [10]. Previous guidelines from the American Dental Association (ADA) and the American Academy of Pediatric Dentistry (AAPD) advocated for an expanded role for dental professionals in HPV vaccination and protection against HPV-OPC. These guidelines emphasized the importance of dental professionals strongly recommending HPV vaccination to all eligible patients and educating both themselves and their patients on the link between HPV and OPC [11,12].

Given the expanding responsibilities of dental professionals in advocating for HPV vaccination and raising awareness about HPV-OPC, it is essential to evaluate their knowledge of HPV and HPV-OPC, their views on their roles in HPV disease prevention, their readiness to engage in discussions regarding HPV vaccination and HPV-OPC as well as the vaccine’s administration, and their actual practices in protecting against HPV-OPC in dental settings [11,12]. Additionally, understanding the perspectives of dental students, who are future dentists, is crucial in this context in promoting primary and secondary interventions. By identifying knowledge gaps, barriers, and attitudes towards preventive measures, targeted interventions can be developed to enhance health outcomes [8]. A previous review was limited to one country and only considered the perspective of dentists [13]. Thus, a comprehensive perspective encompassing a broader range of dental professionals and students is necessary. This study aims to evaluate the knowledge, attitudes, and practices of dental professionals regarding HPV, the association between HPV infection and HPV-OPC, and the HPV vaccine, encompassing its communication and administration in dental settings. The study also captures the perspectives of dental students, who represent the future workforce, in this critical area of healthcare. The goals are to identify areas for enhancement and for the development of targeted interventions to improve the knowledge and confidence among dental practitioners and students regarding HPV vaccine discussion in dental primary healthcare settings, and to provide insights for researchers and policymakers in planning future programs to enhance the readiness of dental professionals to administer HPV vaccines.

## 2. Materials and Methods

### 2.1. Inclusion and Exclusion Criteria

The inclusion criteria for eligible studies included quantitative or qualitative findings regarding the knowledge, perceptions, and behaviors of dental professionals and dental students concerning HPV and HPV-OPC. In this context, dental professionals were defined as dentists and dental hygienists. Perceptions related to HPV and HPV protection were defined as the thought processes concerning education or discussions about HPV, HPV-OPC, or HPV vaccination in dental settings, while behaviors were defined as engaging in actual discussions about HPV, HPV-OPC, or HPV vaccination. Because the first HPV vaccine was introduced in 2006, we included studies published from that year onward [14,15]. Only full-text original articles in English were retrieved and evaluated for inclusion in this study. Grey literature was not included.

The exclusion criteria were as follows: (1) studies that focused solely on knowledge of OPC, without considering the context of HPV, and (2) publications that were editorials, letters, commentaries, or conference abstracts.

### 2.2. Literature Search and Study Selection

We registered this systematic review with PROSPERO (CRD# 42024585928). The Preferred Reporting Items for Systematic reviews and Meta-Analyses (PRISMA) 2020 statement was followed. We searched PubMed/MEDLINE, Embase, and Scopus. The last date of the search was 31 December 2024. The search terms included “human papillomavirus” OR “HPV”, “vaccin*”, “oropharyngeal cancer”, “dentist” OR “dental” OR “oral health” and the corresponding Medical Subject Headings (MeSH) and synonyms. We also manually searched the reference lists to include more eligible articles.

### 2.3. Data Extraction

Two authors (K.C. and T.M.) independently screened the titles and abstracts after removing duplications to identify potentially eligible studies for full-text review. They then screened for full-text relevant studies according to the pre-specified eligibility criteria and further extracted information about the study characteristics (e.g., authors, year, country, study design, population, sample size, sex, age). Any discrepancies between the two authors were resolved through discussion with a senior researcher (H.C.). EndNote was used to manage the references.

### 2.4. Quality Assessment

The Risk of Bias Tool (RoBT) was utilized to assess the risk of bias in all studies included in this review [16]. This tool evaluated 10 items, dividing them into external validity and internal validity measures. Each item in the RoBT was scored as either “0” (indicating the absence of bias) or “1” (indicating the presence of bias), with a total possible score of 10. Based on the RoBT score, studies were categorized as low risk (0 to 3), moderate risk (4 to 6), or high risk (7 to 10) in terms of bias.

## 3. Results

### 3.1. Study Characteristics

**Figure 1:**
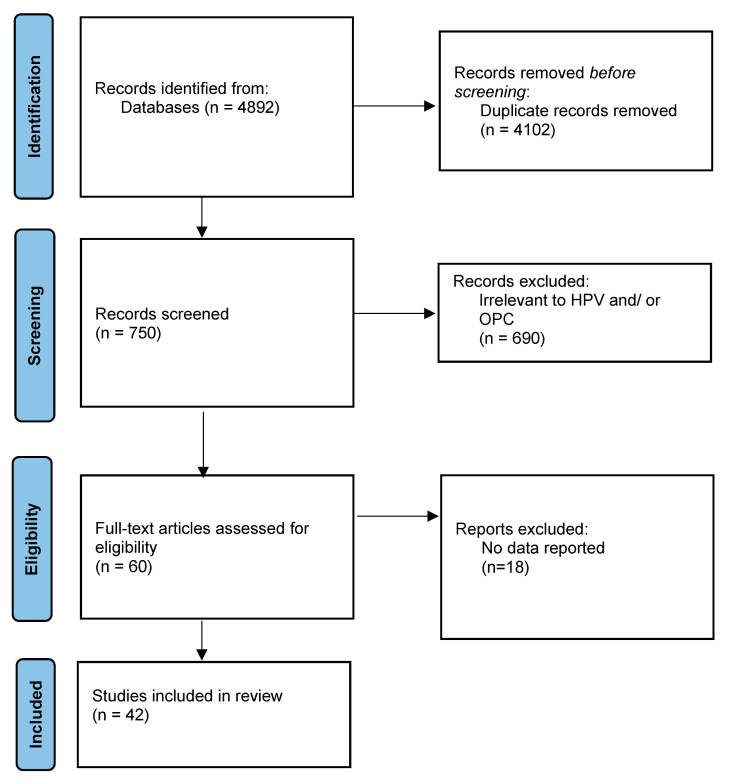
Flow diagram of study selection.

The search yielded 4892 unduplicated articles (Figure 1). We identified 750 records from the initial screening of the titles and abstracts and reviewed 60 reports in full-text form. Forty-two studies were finally deemed eligible for inclusion [17,18,19,20,21,22,23,24,25,26,27,28,29,30,31,32,33,34,35,36,37,38,39,40,41,42,43,44,45,46,47,48,49,50,51,52,53,54,55,56,57,58]. Twenty-three [17,18,19,20,21,22,23,24,25,26,27,28,29,30,31,32,33,34,35,36,37,38,39] and 19 [40,41,42,43,44,45,46,47,48,49,50,51,52,53,54,55,56,57,58] studies evaluated the perceptions of dental practitioners and dental students, respectively. Four [23,42,45,57], five [27,40,41,47,53], and five [46,49,50,53,57] studies covered countries in Europe, Asia, and the Middle East, respectively, while the remaining covered those in the Americas (USA, Canada, and Mexico). Table 1 summarizes the main characteristics of the included studies.

Table 2 summarizes the quality assessment for each study included in this review. All studies had a low risk of bias. None of the studies received financial support from pharmaceutical companies.

### 3.2. Dental Practitioners’ and Students’ Knowledge of HPV

The studies pertaining to the HPV-related knowledge of dental practitioners and dental students were classified into three categories: (i) general HPV information, (ii) the association between HPV and OPC, and (iii) the HPV vaccine.

#### 3.2.1. General HPV Knowledge

According to studies that examined general HPV knowledge, over 90% of dental practitioners were aware that HPV is a sexually transmitted infection [21,22,26,27,29,32] and that engaging in oral sex can be a possible mode of transmission [26]. Additionally, they were able to accurately recognize that HPV infection can cause abnormal pap smear results and may ultimately lead to the development of cervical cancer [22]. Most of them (89–98%) also recognized that HPV infection often has no visible signs or symptoms [29,32]. Studies have identified deficiencies in the knowledge of dental practitioners regarding the epidemiology of HPV infection, as well as the fact that HPV affects both men and women [22,26]. Furthermore, one study reported that dental practitioners have limited knowledge of HPV’s curability and prognosis [22]. The overall knowledge of HPV among dentists and dental hygienists was found to be similar [22,29].

Similarly, studies demonstrated that dental students showed excellent knowledge of HPV as a sexually transmitted infection [40,45,46,50,51,52,55,58] and its link to cervical cancer [40,48,51,52,55,57,58]. Most dental students ware aware that HPV infection often does not have visible signs or symptoms [40,45,51,58] and can affect both genders [51].

#### 3.2.2. Association Between HPV and OPC

Studies that evaluated the knowledge of dental practitioners regarding the link between HPV and OPC showed an improvement in knowledge over the years. While an earlier qualitative study published in 2011 [17] found that the baseline knowledge of this link was relatively low, more recent studies that were published after 2017 indicated that most dental practitioners had a better understanding of the link and were aware of the increased incidence of OPC in younger age groups [20,21,26,27,29,30,32]. Despite the improvement in knowledge, a significant proportion of dental practitioners still held the incorrect belief that the risk of HPV-related OPC was higher in females [20,29] and the principal site for HPV-related OPC was the tongue [27,29,32]. It should be noted that OPC typically occurs in various locations within the oropharynx, including the base of the tongue, pharyngeal tonsils, anterior and posterior tonsillar pillars, glossotonsillar sulci, anterior surface of the soft palate and uvula, and lateral and posterior pharyngeal walls [59,60]. The majority of HPV-OPC cases originate from the tonsils [59,60].

Regarding the knowledge of dental students, over 90% of dental students were aware of the association between HPV and OPC [45,48,50,54,55,57,58]. However, similarly to dental practitioners, dental students also showed common misconceptions about the risk in both genders [55] and the principal site for HPV-related OPC being the tongue [46].

#### 3.2.3. HPV Vaccine

Studies have revealed that most dental practitioners are knowledgeable about the existence of a vaccine against HPV. However, some studies have identified gaps in their knowledge about HPV vaccines, such as a lack of awareness of the vaccine’s protection against a growing number of HPV strains, the vaccination recommendations for specific age groups and genders, and the names and availability of marketed HPV vaccines [21,22,24,26].

Studies examining HPV vaccine knowledge among dental students have yielded varied results. One study in the Middle East reported poor overall knowledge among dental students regarding HPV, but did not provide specific details or demonstrations of the areas where knowledge was lacking [49]. Some studies have found that dental students are aware of the availability of a vaccine for the prevention of HPV infection [45,48,50,52], while others have reported that a significant proportion of students had no knowledge about the vaccine [46,50,57]. Knowledge gaps among students include those regarding vaccination recommendations for specific genders [52,54], the dosage [52], and insurance coverage [48,52,58].

### 3.3. Dental Practitioners’ and Students’ Attitudes Towards HPV Vaccine Communication in a Dental Setting

Several articles examined the involvement of dental practitioners in preventing HPV-related OPC [17,19,22,32,37]. Four studies surveyed practicing dentists and hygienists and produced varying results. One study found mixed opinions on whether or not dental professionals should discuss the link between HPV and OPC and the importance of HPV vaccination [23], while the other three found that most professionals believed that they had a role in HPV-OPC prevention [17,32,37]. In two studies, dental practitioners felt that their dental organizations and profession should inform the public about the HPV-OPC connection and vaccination [22,37]. Some studies have shown positive attitudes among dentists in recommending HPV vaccination to their patients or referring them to other non-dental primary healthcare institutions for vaccination [32,37]. In a study involving a pediatric setting, over half of the pediatric dentists believed that it was their responsibility to talk about HPV vaccination. In addition, most thought that discussing sexual health and its association with OPC was part of their pediatric dentistry practice [20].

Some educational interventions, such as short lectures with an educational HPV toolkit containing talking tips for dental practitioners, brochures for patients, and promotional leaflets, were conducted to enhance the discussion of HPV and vaccine recommendations by dental practitioners. After the intervention, most of the participants felt that the program was effective in clarifying their role in educating patients on HPV [25,30,31].

Despite the overall positive perception, small portions showed negative concerns over HPV vaccine communication in the dental setting. Other than low comfortability in discussing the HPV vaccine with patients [29,30], some dentists believed that medical professionals were the only appropriate source of information regarding HPV vaccination [19,29].

In dental schools, the majority of dental students believed that discussing and recommending HPV vaccines fell within the scope of dental practice [44,55,57]. Furthermore, senior dental students were more likely to have positive attitudes towards HPV vaccine communication than their junior counterparts [53]. However, the major concern for dental students is their lack of confidence in discussing and recommending the HPV vaccine to their patients [44,55].

### 3.4. Dental Practitioners’ Practices in HPV Vaccine Communication in a Dental Setting

Studies have indicated that dental practitioners do not adequately communicate with their patients about HPV vaccination [19,20,21,22,26,29,37]. Stull et al. demonstrated that HPV vaccination was discussed by only 9% of dental practitioners [29]. Dentists and dental hygienists were found to be equally deficient in this regard [22]. While many practitioners acknowledged the importance of discussing HPV vaccination with their patients [17,20,21,23,25,26], they also expressed concerns about the challenges associated with such discussions [19,20,21,23,24,26].

In contrast to current dental practitioners, a considerable proportion of dental students (40–80%) have expressed their willingness to discuss or recommend HPV vaccination to their patients in their future practice [44,49,52].

### 3.5. Dental Practitioners’ and Students’ Barriers to HPV Vaccine Communication in a Dental Setting

The studies identified various factors that dental practitioners perceive as challenging when discussing HPV-related information with their patients. A significant challenge that dental practitioners and dental students faced when discussing HPV-related information with their patients was their discomfort or lack of confidence in doing so. This discomfort is often linked to the social pressure that dental practitioners may encounter in the communities that they serve and the perceived patients’ resistance to the discussion. These factors can make it difficult for them to have open and effective conversations about HPV vaccination with their patients [17,18,29,30,44,55]. Two studies found that dental practitioners varied in their likelihood to counsel different patient populations about HPV vaccination [21,26]. Greater difficulties were highlighted when discussing corresponding information with the elderly. To overcome the challenges in discussing HPV-related information with patients, some studies have proposed educational strategies to improve dental practitioners’ knowledge, ability to provide counseling, and self-efficacy [17,23,25,30,31]. Cotter et al. reported that students’ knowledge and confidence in discussing HPV, HPV-related OPC, and HPV immunization increased after completing a training module covering knowledge and practical skills, suggesting that effective education and preparation for HPV counseling can be accomplished through modular-based education in the dental curriculum [48].

Other barriers to effective HPV communication and interaction between dental practitioners and patients include concerns about practitioners’ ages, the perceived role of a dentist, leadership dynamics, patient privacy, and time constraints. These factors may hinder effective communication about HPV and make it difficult for providers to address related topics with patients [19,20,21,23,24,26,28,33,34,35,36,37,38].

### 3.6. Dental Practitioners’ and Students’ Perceptions of HPV Vaccine Administration in a Dental Setting

Dentists maintained a mostly neutral stance, while hygienists expressed disagreement regarding the administration of the HPV vaccine within their professional scope [32,35,39]. A study by Guadiana revealed that 51% of dentists and 63% of hygienists would be willing to administer the HPV vaccine if it was legally permitted [35]. However, they expressed lower confidence in carrying out this task due to barriers such as liability concerns and insufficient knowledge [32,35]. Despite these challenges, both dentists and hygienists expressed a willingness to undergo training programs to facilitate the promotion and administration of the HPV vaccine in their practices [32].

Two studies indicated that the significant majority (82%) of dental students expressed support for dentists administering the HPV vaccine in their future practice [35,55]. However, conflicting results were reported in another study [44]. Similarly to dental providers, the major barriers identified by dental students in administering the HPV vaccine in their future practice included a lack of knowledge and liability concerns [35]. Additionally, 80% of dental students expressed a greater willingness to administer the HPV vaccine if they received proper training [52].

## 4. Discussion

Dental practitioners have long been recognized as key players in promoting primary healthcare and preventive services. Their involvement in providing dietary advice and smoking cessation programs is a testament to their vital role in public health [5,6,7,8]. In the US, guidelines have been issued to expand the role of dental professionals in HPV vaccination and OPC prevention: the ADA and AAPD recommend that dentists support the use and administration of the HPV vaccine when counseling their patients [4,11,12]. While the specific guidelines may vary across countries, promoting health, educating patients on the prevention and treatment of oral diseases, and supporting systemic and oral health maintenance are fundamental competencies expected of dental professionals worldwide, such as in Hong Kong [61]. In the US, dentists in states like Oregon are allowed to administer all vaccines, including the HPV vaccine [35]. Similar regulations exist in Minnesota and Illinois, as well as in countries like Scotland, reflecting the growing awareness of the importance of vaccination and preventive healthcare practices [35]. This further underscores the expanding role of dental professionals in promoting overall health and well-being. However, the current literature reports mixed findings on the knowledge, attitudes, and practices of dentists concerning HPV vaccination discussions and administration in dental settings [13]. To our knowledge, this study is the first to provide a comprehensive and up-to-date review of the literature on the role of dental professionals in HPV vaccination discussions in dental settings, incorporating the perspectives of current practitioners and dental students, who are the future workforce in dentistry. Thus, this review sheds light on effective ways in which dental professionals can contribute to the prevention of HPV-related OPC.

Studies have shown that there is suboptimal communication regarding HPV-OPC and HPV vaccination in dental settings among practicing dental professionals [19,20,21,22,23,24,26,29,37], while dental students are more willing to engage in these discussions in their future practice [44,49,52]. It is crucial to overcome barriers to practicing effective communication. One common barrier is the discomfort and lack of confidence that dental professionals perceive when discussing HPV vaccination [17,18,29,30,44,55]. A major reason for this is a knowledge deficiency, such as a lack of awareness that HPV can also affect men, that the HPV vaccine is recommended for men, and that the incidence of HPV-related OPC in men is increasing [20,29]. Therefore, there is a need to improve the knowledge and thus confidence levels among dental professionals to ensure effective communication and increase the uptake of HPV vaccination.

There is an increasing focus in public health on the significance of evidence-based approaches to enhance the health of the population [62]. While few theoretical frameworks have been developed in the past, it is encouraging to see an increasing number of educational interventions being implemented in different studies, particularly among dental students, who are future dental professionals, to improve OPC and HPV education, including knowledge, attitudes, and practices [25,30,31,48,51,52,56]. These interventions provide valuable insights for the integration of oral cancer- and HPV-related topics into dental curricula for students. However, the current dental practitioners have lacked training in this area in their past curricula, although there are now several education interventions available for them. Scholarly journals and continuing education courses have been identified as primary sources of HPV information for dental professionals, underscoring the significance of integrating HPV topics into professional meetings and lectures to meet continuing education requirements [13]. This integration aims to enhance healthcare providers’ knowledge, skills, and self-efficacy in effectively addressing HPV-related issues. Hands-on training workshops can further facilitate skill-building and address barriers to HPV prevention by improving the communication skills among providers. Emphasizing primary prevention efforts, such as patient education on HPV, recommending the HPV vaccine, and actively referring patients for vaccination, dental providers can play a pivotal role in HPV prevention. Professional organizations can also support these endeavors by disseminating scientific information, providing clinical guidelines, and promoting inter-professional collaboration with disciplines like public health and medicine, thereby strengthening HPV prevention strategies across healthcare sectors.

Effective communication channels are a crucial component in improving dental practitioners’ self-efficacy in discussing this topic and in promoting HPV vaccination in dental settings. The previous literature suggests passive communication methods, such as providing written fact sheets, parent education websites, and HPV vaccine decision aids, as helpful tools to initiate discussions [22,63]. Additionally, technology, such as short videos for waiting rooms and text messages, can enhance communication and has shown success in promoting public health [13,23]. The use of chat-based instant messaging support for smoking cessation interventions is an example of a successful measure in primary healthcare settings [64,65]. This approach has been shown to be effective in helping individuals to quit smoking and may also be considered in promoting HPV-OPC education and HPV vaccination in dental settings. Further research is necessary to identify the most efficient print and technology communication channels to enhance the communication skills among dental providers. Tailored communication strategies for different patient subgroups, particularly the elderly or children, who may face greater challenges in discussing sensitive topics such as HPV vaccination, should also be explored [21,26]. By establishing effective communication channels that take into account patient preferences and needs, dental practitioners can contribute to increased HPV vaccination uptake in their patients.

As outlined by the current dental professionals and dental students, liability is a primary concern for them when administrating the HPV vaccine in their dental offices [32,35]. Since administering HPV vaccines is not currently a standard practice, enhancing the willingness of dental professionals to engage in vaccine-related activities would necessitate clear endorsement by professional organizations for the administration of the HPV vaccine in dental settings. This aligns with previous studies highlighting the importance of strong support from professional dental associations in promoting and enabling dental professionals in HPV patient education [23,37]. Given that dentists and dental students are generally willing to administer the HPV vaccine if adequately trained, offering practical HPV vaccine administration training during work and in dental schools may enhance the confidence and readiness of these existing and upcoming dental professionals to vaccinate patients in the dental setting [32,52].

Safe and effective HPV vaccines have been available for girls since 2006 and for boys since 2011 to prevent HPV-associated cancers [59,60]. Initially recommended for cervical cancer, the US Food and Drug Administration expanded its approval of the HPV vaccine in 2020 to include the prevention of HPV-OPC [59,60]. Previous studies have demonstrated that HPV vaccination, particularly in males, who are predominantly affected by HPV-OPC, can provide protection against HPV oncogenic types 16/18 infection and could help in the prevention of the disease [66,67]. This highlights the significant role that pan-gender HPV vaccination could play in the future battle against OPC. However, due to the recent adoption of male HPV vaccination and the delayed onset of the disease, the long-term effects of male HPV vaccination on the OPC incidence remain to be seen over the next 30–40 years [68]. Further well-designed studies investigating the effects of HPV vaccination in men and its impact on the development of OPC would be particularly beneficial.

This study has several limitations. Firstly, the results may have been influenced by the cross-sectional design and the self-reported measures used to assess providers’ knowledge and perceptions of HPV and discussions about HPV protection. Secondly, the fact that most of the studies were from Western countries may limit the generalizability of our findings to regions like Asia with different cultural backgrounds. Despite our inability to conduct a meta-analysis due to the lack of homogenous and standardized data across the included studies, our study offers an up-to-date and comprehensive understanding of both dental professionals and students, which has important implications for the current state of HPV vaccination discussions in dental settings.

## 5. Conclusions

This study identified gaps in knowledge and confidence among dental practitioners and students regarding HPV vaccination, with limited discussion of the topic in dental settings and varying attitudes towards administering the vaccine. Evidence from this study can be used to identify strategies to improve the knowledge and confidence among dental practitioners and students regarding HPV vaccine discussion in dental primary healthcare settings. It also provides insights for researchers and policymakers in planning future programs to enhance the readiness of dental professionals to administer HPV vaccines.

## Figures and Tables

**Table 1:** Study characteristics.

Study	Country	Study Design	Population	Sample Size	Findings/Conclusions
Dental Practitioners					
Americas (USA, Canada, and Mexico)					
Daley et al. (2011) [17]	USA	Focus group interview	Dentists, dental hygienists	38	Effective and comfortable communication is required regarding the HPV-OPC link and the potential uses of HPV vaccines.
Shepperd et al. (2013) [18]	USA	Cross-sectional survey	Dentists	929	Parent/public approvals were the primary barriers to HPV counseling.
Daley et al. (2014) [19]	USA	Cross-sectional survey	Dentists	210	Findings suggest liability and perceived role in processes of change necessary to guide dentists to primary prevention of HPV-related OPC despite high levels of knowledge.
Hosking et al. (2017) [20]	USA	Cross-sectional survey	Dentists	64	The majority of dentists believe that they should be discussing the human papillomavirus vaccine with patients and parents.
Thompson et al. (2017) [21]	USA	Focus group interview	Dental hygienists	48	Dental hygienists recognized the importance of HPV and OPC prevention efforts, including the promotion of the HPV vaccine.
Daley et al. (2018) [22]	USA	Cross-sectional survey	Dentists, dental hygienists	182	HPV knowledge deficits among dental providers were identified.
Kline et al. (2018) [23]	USA	Mixed method: Focus group interview, cross-sectional survey	Dentists, dental hygienists	284	There is a need for dental providers to follow professional organizations’ guidance to advance prevention efforts and reduce the HPV-related cancer incidence.
Naleway et al. (2018) [24]	USA	Cross-sectional survey	Dentists, dental hygienists, dental assistants	234	Dental providers were less comfortable making vaccine recommendations to their patients. Vaccine recommendations are not a traditional practice among dental providers and may require additional education and communication tools.
Shukla et al. (2018) [25]	USA	Cross-sectional survey	Dentists, dental hygienists	40	HPV educational intervention was well received and successful in improving the self-reported knowledge, comfort levels, and preparedness of dental providers in discussing HPV with their patients.
Vázquez-Otero et al. (2018) [26]	USA	Focus group interview	Dentists	33	Creating awareness of trusted informational sources, as well as increasing HPV knowledge and understanding the multiple patient and practice appraisal factors, is required. Enhancing the communication skills of dentists with patients is needed to improve HPV-related cancer prevention education.
Griner et al. (2019) [28]	USA	Focus group interview	Dental professionals	13	Barriers included HPV as a sensitive topic and the need for HPV-related education and skills. Facilitators included perceptions of HPV prevention as part of the dental provider’s role and the potential development of passive educational methods to provide HPV-related information to patients.
Stull et al. (2019) [29]	USA	Cross-sectional survey	Dentists, dental hygienists	318	Barriers preventing dental providers from discussing the HPV vaccine with patients, including a lack of knowledge and discomfort in discussing a sexually transmitted infection.
Harris et al. (2020) [39]	USA	Cross-sectional survey	Dentists	203	The majority of Utah dentists support the role of HPV education with direct patient counseling and brochures but are not interested in providing the vaccine.
Salous et al. (2020) [30]	USA	Cross-sectional survey	Dentists, dental hygienists, dental assistants, dental therapists	122	An educational intervention was effective in improving OHPs’ knowledge of HPV and enhancing their comfortability and preparedness to discuss vaccination with their patients.
Pampena et al. (2020) [31]	USA	Cross-sectional survey	Dentists, dental hygienists, dental assistants	263	Educational lectures can be effective in increasing dental providers’ knowledge and awareness about HPV, HPV-related cancers, and vaccination.
Patel et al. (2020) [32]	USA	Cross-sectional survey	Dentists, dental hygienists	711	Most respondents answered HPV knowledge questions correctly but did not know that HPV-related OPC has a more favorable prognosis than other head and neck cancers. Dentists were more confident discussing and recommending the HPV vaccine. Both dentists and hygienists were willing to refer patients to their non-dental primary care providers for vaccination.
Arnell et al. (2021) [33]	USA	Cross-sectional survey	Dentists, dental hygienists	266	Dentists were more knowledgeable about HPV vaccination and more likely to recommend the vaccine than hygienists. Higher levels of HPV-related knowledge correlated positively with beliefs and practices that support HPV vaccine advocacy.
Askelson et al. (2021) [34]	USA	Mixed methods: qualitative interview, cross-sectional survey	Dentists, dental hygienists	509	Dental providers expressed a willingness to participate in HPV vaccine promotion, and future efforts should focus on addressing barriers to doing so.
Guadiana et al. (2021) [35]	USA	Cross-sectional survey	Dentists, dental hygienists, dental students, dental hygiene students	623	Dental professionals were variably confident discussing HPV with patients and generally believed that it enhanced patients’ health. Stronger confidence and beliefs were associated with greater willingness to administer the vaccine. Barriers among professionals opposing the HPV vaccine included a lack of knowledge of the subject, liability concerns, and personal beliefs.
Askelson et al. (2023) [36]	USA	Mixed methods: qualitative interview, cross-sectional survey	Dentists, dental hygienists	509	Knowledge was identified as a key barrier to providing a strong recommendation for HPV vaccination, and convenience was the most important factor to consider for any future continuing education.
Coyne et al. (2023) [37]	Canada	Cross-sectional survey	Dentists	196	Most agreed that discussing the link between HPV and oropharyngeal cancer falls within their scope of practice. Barriers that may contribute to this unwillingness include a lack of professional policies and guidelines.
JaKa et al. (2024) [38]	USA	Semi-structured qualitative interview	Dental practitioners	11	Although most dental providers thought that HPV vaccination was important, they lacked detailed knowledge about when and to whom the vaccine should be recommended.
**Asia**					
Arora et al. (2018) [27]	Malaysia	Cross-sectional survey	Dentists	179	A substantial increase in awareness is required among dental professionals regarding the HPV-OPC link.
**Dental students**					
**Americas (USA, Canada, and Mexico)**					
Rutkoski et al. (2018) [43]	USA	Cross-sectional survey	Dental students, dental hygiene students	46	Dental students’ knowledge of HPV, HPV-OPC, and the HPV vaccine was satisfactory.
Kepkaa et al. (2019) [44]	USA	Cross-sectional survey	Dental students, dental hygiene students	306	The major barrier to engaging oral health students in HPV vaccination efforts was role conflict.
Cotter et al. (2020) [48]	USA	Pre–post testing	Dental hygiene students	40	The students demonstrated an increase in confidence and comfort in providing HPV immunization counseling on the post-test, as well as an increase in positive attitudes about recommending HPV immunization.
Stull et al. (2021) [51]	USA	Pre–post testing	Dental students, dental hygiene students	57	Results of this study suggest that dental hygiene education programs should include didactic instruction on HPV, the use of brief motivational interviewing (BMI) strategies, and multiple opportunities to practice HPV-related conversations to improve student knowledge, attitudes, comfort, and confidence levels. Interactive continuing education programs with a focus on HPV and BMI techniques can also assist oral healthcare providers in the delivery of provider–patient communication on HPV.
Wright et al. (2021) [52]	USA	Cross-sectional survey	Undergraduate dental students	173	Most respondents did not know the prevalence of HPV and the prognosis of HPV-OPC.
Pinzon et al. (2022) [54]	USA, Mexico	Cross-sectional survey	Dental students, dental hygiene students	114	Dental students’ knowledge of HPV, HPV-OPC, and the HPV vaccine was satisfactory.
Torres et al. (2022) [55]	USA	Cross-sectional survey	Dental students	109	Most dental students believed that HPV prevention was within their scope of practice; however, half reported feeling somewhat/not at all confident in recommending the vaccine and performing oral cancer exams.
Thanasuwat et al. (2023) [56]	USA	Cross-sectional survey	Medical and dental trainees	74	The proposed online educational intervention was effective in improving HPV-related cancer and HPV vaccine knowledge, as well as attitudes towards vaccine recommendation, among dental and medical trainees.
**Asia**					
Doshi et al. (2015) [40]	India	Cross-sectional survey	Dental students	233	A lack of awareness with regard to HPV among students was recognized.
Rajiah et al. (2017) [41]	Malaysia	Cross-sectional survey	Undergraduate dental students	142	Dental students’ knowledge of HPV had no influence on their attitudes towards HPV vaccines.
Balaji et al. (2020) [47]	India	Cross-sectional survey	Undergraduate dental and medical students	577	The dental undergraduate students presented an average level of knowledge and awareness regarding HPV and its prevention.
**Middle Eastern regions**					
Sallam et al. (2019) [46]	Jordan	Cross-sectional survey	Doctoral students	376	Gaps in knowledge regarding HPV-related oral cancer have been detected, which necessitate intervention measures including curricular changes, training workshops, and awareness campaigns.
Farsi et al. (2020) [49]	Saudi Arabia	Cross-sectional survey	Undergraduate dental students	500	Knowledge about HPV, its vaccine, and HPV-related OPC was low among a sample of Saudi undergraduate dental students.
Keser et al. (2021) [50]	Turkey	Cross-sectional survey	Dental students	318	The majority of the study participants had some baseline understanding of HPV prior to accessing the modules. After reviewing the modules, there was a statistically significant increase in the proportion of respondents who identified OPC as associated with HPV.
Lingam et al. (2022) [53]	Egypt, India, Pakistan, Saudi Arabia, UAE, and Sudan	Cross-sectional survey	Undergraduate students	886	Disparities in knowledge regarding HPV-related oral cancer have been detected among female and male participants in different nations.
Alsharif et al. (2024) [57]	Saudi Arabia	Cross-sectional survey	Undergraduate dental students	453	A significant knowledge gap was observed, including regarding the common sites of HPV-OPC. The participants showed a favorable attitude towards their responsibility of informing patients about HPV-OPC and advocating for HPV immunization.
**Europe**					
Rakhra et al. (2018) [42]	UK	Cross-sectional survey	Undergraduate dental students	211	Dental students demonstrated an understanding of HPV and OPC and expressed that they felt dentists should play a role in health promotion in relation to OPC.
Lorenzo-Pouso et al. (2019) [45]	Spain	Cross-sectional survey	Dental students	158	Most students believed that HPV infection was linked to oropharyngeal cancer.
Poelman et al. (2024) [58]	Netherlands	Cross-sectional survey	Dental hygiene students	232	Deficits in the knowledge of HPV and HPV vaccination were observed among Dutch dental hygiene students.

**Table 2:** Quality assessment.

Risk of Bias Items	External Validity				Internal Validity						Overall Risk of Study Bias
	1	2	3	4	5	6	7	8	9	10	
Daley et al. (2011) [17]	No	Yes	Yes	Yes	Yes	Yes	No	Yes	Yes	Yes	Low, 2
Shepperd et al. (2013) [18]	Yes	Yes	Yes	Yes	Yes	Yes	Yes	Yes	Yes	Yes	Low, 0
Daley et al. (2014) [19]	Yes	Yes	Yes	Yes	Yes	Yes	Yes	Yes	Yes	Yes	Low, 0
Hosking et al. (2017) [20]	Yes	Yes	Yes	Yes	Yes	Yes	Yes	Yes	Yes	Yes	Low, 0
Thompson et al. (2017) [21]	No	Yes	Yes	Yes	Yes	Yes	No	Yes	Yes	Yes	Low, 2
Daley et al. (2018) [22]	Yes	Yes	Yes	Yes	Yes	Yes	Yes	Yes	Yes	Yes	Low, 0
Kline et al. (2018) [23]	No	Yes	Yes	Yes	Yes	Yes	No	Yes	Yes	Yes	Low, 2
Naleway et al. (2018) [24]	Yes	Yes	No	Yes	Yes	Yes	Yes	Yes	Yes	Yes	Low, 1
Shukla et al. (2018) [25]	Yes	Yes	Yes	Yes	Yes	Yes	Yes	Yes	Yes	Yes	Low, 0
Vázquez-Otero et al. (2018) [26]	No	Yes	Yes	Yes	Yes	Yes	No	Yes	Yes	Yes	Low, 2
Arora et al. (2018) [27]	Yes	Yes	Yes	Yes	Yes	Yes	Yes	Yes	Yes	Yes	Low, 0
Griner et al. (2019) [28]	No	Yes	Yes	Yes	Yes	Yes	No	Yes	Yes	Yes	Low, 2
Stull et al. (2019) [29]	No	Yes	Yes	Yes	Yes	Yes	No	Yes	Yes	Yes	Low, 2
Salous et al. (2020) [30]	Yes	Yes	No	Yes	Yes	Yes	Yes	Yes	Yes	Yes	Low, 1
Pampena et al. (2020) [31]	Yes	Yes	Yes	Yes	Yes	Yes	Yes	Yes	Yes	Yes	Low, 0
Patel et al. (2020) [32]	Yes	Yes	Yes	Yes	Yes	Yes	Yes	Yes	Yes	Yes	Low, 0
Arnell et al. (2021) [33]	Yes	Yes	No	Yes	Yes	Yes	Yes	Yes	Yes	Yes	Low, 1
Askelson et al. (2021) [34]	No	Yes	Yes	Yes	Yes	Yes	No	Yes	Yes	Yes	Low, 2
Guadiana et al. (2021) [35]	Yes	Yes	Yes	Yes	Yes	Yes	Yes	Yes	Yes	Yes	Low, 0
Askelson et al. (2023) [36]	No	Yes	Yes	Yes	Yes	Yes	No	Yes	Yes	Yes	Low, 2
Coyne et al. (2023) [37]	Yes	Yes	Yes	Yes	Yes	Yes	Yes	Yes	Yes	Yes	Low, 0
JaKa et al. (2024) [38]	No	Yes	Yes	Yes	Yes	Yes	No	Yes	Yes	Yes	Low, 2
Harris et al. (2020) [39]	No	Yes	Yes	Yes	Yes	Yes	No	Yes	Yes	Yes	Low, 2
Doshi et al. (2015) [40]	Yes	Yes	Yes	Yes	Yes	Yes	Yes	Yes	Yes	Yes	Low, 0
Rajiah et al. (2017) [41]	Yes	Yes	No	Yes	Yes	Yes	Yes	Yes	Yes	Yes	Low, 1
Rakhra et al. (2018) [42]	Yes	Yes	Yes	Yes	Yes	Yes	Yes	Yes	Yes	Yes	Low, 0
Rutkoski et al. (2018) [43]	Yes	Yes	Yes	Yes	Yes	Yes	No	Yes	Yes	Yes	Low, 1
Kepkaa et al. (2019) [44]	Yes	Yes	Yes	Yes	Yes	Yes	Yes	Yes	Yes	Yes	Low, 0
Lorenzo-Pouso et al. (2019) [45]	Yes	Yes	Yes	Yes	Yes	Yes	Yes	Yes	Yes	Yes	Low, 0
Sallam et al. (2019) [46]	Yes	Yes	Yes	Yes	Yes	Yes	Yes	Yes	Yes	Yes	Low, 0
Balaji et al. (2020) [47]	Yes	Yes	Yes	Yes	Yes	Yes	Yes	Yes	Yes	Yes	Low, 0
Cotter et al. (2020) [48]	No	Yes	Yes	Yes	Yes	Yes	No	Yes	Yes	Yes	Low, 2
Farsi et al. (2020) [49]	Yes	Yes	Yes	Yes	Yes	Yes	Yes	Yes	Yes	Yes	Low, 0
Keser et al. (2021) [50]	Yes	Yes	Yes	Yes	Yes	Yes	Yes	Yes	Yes	Yes	Low, 0
Stull et al. (2021) [51]	No	Yes	Yes	Yes	Yes	Yes	No	Yes	Yes	Yes	Low, 2
Wright et al. (2021) [52]	Yes	Yes	Yes	Yes	Yes	Yes	Yes	Yes	Yes	Yes	Low, 0
Lingam et al. (2022) [53]	Yes	Yes	Yes	Yes	Yes	Yes	Yes	Yes	Yes	Yes	Low, 0
Pinzon et al. (2022) [54]	Yes	Yes	Yes	Yes	Yes	Yes	Yes	Yes	Yes	Yes	Low, 0
Torres et al. (2022) [55]	Yes	Yes	Yes	Yes	Yes	Yes	Yes	Yes	Yes	Yes	Low, 0
Thanasuwat et al. (2023) [56]	Yes	Yes	Yes	Yes	Yes	Yes	Yes	Yes	Yes	Yes	Low, 0
Alsharif et al. (2024) [57]	Yes	Yes	Yes	Yes	Yes	Yes	Yes	Yes	Yes	Yes	Low, 0
Poelman et al. (2024) [58]	Yes	Yes	Yes	Yes	Yes	Yes	Yes	Yes	Yes	Yes	Low, 0

## Data Availability

No new data were created or analyzed in this study. Data sharing is not applicable to this article.
